# How can patients shape digital medicine? A rapid review of patient and public involvement and engagement in the development of digital health technologies for neurological conditions

**DOI:** 10.1080/14737167.2024.2410245

**Published:** 2024-10-07

**Authors:** Megan Hanrahan, Cameron Wilson, Alison Keogh, Sandra Barker, Lynn Rochester, Katie Brittain, Jack Lumsdon, Ríona McArdle

**Affiliations:** a Population Health Sciences Institute, Newcastle University, Newcastle, UK; b School of Clinical Medicine, Department of Public Health and Primary Care, University of Cambridge, Cambridge, UK; c School of Medicine, Trinity College Dublin, Dublin, Ireland; d Public Patient Advisory Group, Newcastle University, Newcastle, UK; e Translational and Clinical Research Institute, Newcastle University, Newcastle, UK

**Keywords:** Digital health, nervous systems diseases, neurocognitive disorders, patient participation, patient involvement, qualitative research, review

## Abstract

**Introduction:**

Patient and Public Involvement and Engagement (PPIE) involves working ‘*with*’ or ‘*by*’ patients and the public, rather than ‘*to*,’ ‘*about*,’ or ‘*for*’ them, and is integral to neurological and digital health research. This rapid review examined PPIE integration in the development and implementation of digital health technologies for neurological conditions.

**Methods:**

Key terms were input into six databases. Included articles were qualitative studies or PPIE activities involving patient perspectives in shaping digital health technologies for neurological conditions. Bias was evaluated using the NICE qualitative checklist, with reporting following PRISMA guidelines.

**Results:**

2,140 articles were identified, with 28 included. Of these, 25 were qualitative studies, and only three were focused PPIE activities. Patient involvement was mostly limited to one-off consultations during development.There was little evidence of PPIE during implementation, and minimal reporting on its impact.

**Conclusions:**

PPIE has been inconsistently reported in this research area, highlighting the need for more guidance and best-practice examples This review used a UK-based definition of PPIE, which may have excluded relevant activities from other countries. Future reviews should broaden terminology to capture PPIE integration globally.

## 1. Introduction

Neurological conditions, such as Alzheimer’s disease, epilepsy, Parkinson’s disease, and multiple sclerosis, are highly prevalent and have a major impact on individual health and healthcare services. For example, Alzheimer’s disease affects over 55 million people globally and is currently the leading cause of dementia, significantly impairing cognitive function and impacting daily living [1,2]. Epilepsy, which affects over 50 million, is characterized by recurrent seizures that can lead to physical stigma and psychosocial challenges [3]. Parkinson’s disease, affecting more than 10 million people, leads to a variety of motor and non-motor symptoms that significantly impacts one’s quality of life [4,5]. Multiple sclerosis affects over 2.8 million people and results in a range of neurological impairments [6]. These conditions pose significant burdens on patients and present significant challenges to healthcare systems, highlighting the need for innovative solutions to improve monitoring, treatment, and patient outcomes [7,8].

To evaluate a person’s health, examining specific criteria can help to establish the condition and progression of an illness. These criteria, known as clinical outcomes, encompass parameters used to understand certain aspects of health and any changes because of an intervention [9]. Recent technological innovations now enable us to observe these outcomes remotely through digital health technologies [10]. Tracking health digitally can be particularly useful for people with neurological conditions, which are often progressive and require adjustments to treatment and care and could therefore benefit from remote monitoring [11]. Digital health technologies can help clinicians gather a more comprehensive and dynamic understanding of a patient’s health status by capturing clinically meaningful measurements continuously in real-life situations [12]. This, in turn, could improve the efficiency and efficacy of disease management [12].

Digital health technologies have generated enthusiasm in a clinical setting, as they offer the potential of a more accurate evaluation of a patient’s health journey [13]. Through remote monitoring of real-time activities, previously unknown aspects of disease impact, such as contextual influences, can be unveiled, and this in turn, can significantly reduce drug expense [13]. Digital health technologies can be particularly important in neurological research. For example, people with Parkinson’s disease often report unique and unmet healthcare needs [14,15]. Unmet healthcare needs included timely diagnoses and treatment, access to specialized care, and access to information and education. Traditional methods for assessing Parkinson’s symptoms often fall short in capturing the full patient experience and disease progression accurately [16]. Digital health technologies could help bridge this gap and address certain unmet healthcare needs by enabling more frequent monitoring. For instance, common motor symptoms in people with PD can be objectively measured using digital technology, including data in smartphones or inexpensive sensors [17,18], and recent technological advancements include infrared cameras and accessible accelerometers, utilizing an array of sensors [19]. Accelerometer data gives clinicians a clearer understanding of the patient experience, and thereby encouraging the creation of more tailored and relevant treatment plans [20]. This echoes for other neurological conditions, such as multiple sclerosis or muscular dystrophy; a more comprehensive understanding of digitally derived outcomes, such as physiological and cognitive measures, is fundamental in assigning appropriate treatment plans [21].

To fully realize these advantages, it is important to dedicate effort to both technical validation of digital health technologies and to fostering an environment that supports their creation and implementation. Therefore, while we can recognize the benefits of sensor and data-based approaches in delivering effective healthcare as researchers, these advantages are only felt if we have the support and trust of the target community across the research process [22]. Alongside professional collaboration, active and frequent patient involvement and engagement are crucial for making thoughtful decisions about what matters most in the development of successful digital health technologies [23]. Further, regulatory bodies, such as the European Medical Association and the Food and Drug Administration, have prioritized patient involvement in the qualification of digital health technologies; they indicate that relevance and meaningfulness to patients are central in any validation [24,25]. Without measurements that are centered around patients, stakeholders run the risk of perpetuating inadequate traditional assessments through digital means and proliferating unproductive tools that hinder both the quality and efficiency of clinical research and care [22].

In response to this need for patient-centered development, the inclusion of PPIE activities in the development and improvement of digital health technologies is becoming increasingly endorsed in health policy and research [26]. The UK Standards for Public Involvement define PPIE as the active involvement of patients, caregivers, and/or the public in the research process [27]. The National Institute for Health and Care Research (NIHR) clearly defines PPIE as work that is done ‘*with*’ or ‘*by*’ patients and the public rather than ‘*to*,’ ‘*about*,’ or ‘*for*’ them, and emphasizes the importance of involving contributors across the entire research cycle, from identifying the research question to implementation [27]. We use the term ‘contributors’ throughout to refer to individuals who offer insights from a PPIE perspective. Involving contributors in digital health research is encouraged to improve relevance, quality, and impact for the end users [27]. This includes tasks such as translating technical publications to plain English for a broader audience, assessing the practicality of digital tools in the real-world, and helping place research in a wider context. Although important, there is no widely accepted PPIE structure that can be applied to all research programs. Health-based research is often context specific and despite strong encouragement to include PPIE in neurological research, and an abundance of advantages to embed the patient's voice in the process, reporting of this work is either inconsistent or remains unclear, missing, or lacking representation [28–31].

A few reviews have reported on PPIE activities. For example, Gray et al. [32] identified from 89 randomized control trials no evidence of PPIE activities in nurse science journals, Owyang et al. [33] identified only two orthopedic trials out of 475 that evidenced purposeful PPIE, and Price et al. [34] identified an increase to 11% frequency of authors reporting PPIE in general medicine research from 2014–2018. All indicate infrequent involvement. Another review by Rouncefield-Swales et al. [35] that focused on children’s and young people’s PPIE contributions in 40 health-related papers displayed frequent involvement and good quality reporting, with the majority exclusively reporting on PPIE activities. However, they found that the evaluation of PPIE impact was poorly reported among their search and recommended future improvement. The present rapid review, to our knowledge, will be the first to examine existing PPIE work conducted in digital health research with a focus on neurological conditions.

### 1.1. Research question

To what extent has the integration and impact of PPIE activities had on the development of digital health technologies for neurological conditions, and how effective have these strategies been?

### 1.2. Aim and objectives

The aim of this review is to evaluate the integration and effectiveness of PPIE activities in the development of digital health technologies for neurological conditions. To achieve this there are two objectives:

1. To report on how PPIE activities have been integrated into the development of digital health technologies for neurological conditions.
2. To assess the effectiveness of these strategies, highlighting what we can learn from this work and offer recommendations for future practice.

## 2. Materials and methods

The protocol of this review was registered in PROSPERO (CRD42023428736). A rapid scoping review was considered the most appropriate design given the scope of the research question and aims to provide an overview, identify gaps, and provide recommendations. Further, this streamlined way of synthesis fits the limited timeframe of the current review, allowing us to produce evidence in a timely manner [36]. Therefore, we followed the PRISMA guidelines as a basis for reporting (Appendix B) [37].

### 2.1. Eligibility criteria

Studies were selected based on the following inclusion and exclusion criteria:

#### 2.1.1. Defining PPIE for this review

Included are papers that report on PPIE activities, as defined by the NIHR (i.e. work that is done ‘*with*’ or ‘*by*’ patients and the public rather than ‘*to*,’ ‘*about*,’ or ‘*for*’ them [27]), and qualitative research that looks at improving the service, development, or implementation of digital health technologies. We recognize that qualitative and PPIE activities are distinct, however, because of the lack of evidence exclusively reporting on PPIE activities, as defined by the NIHR, we broadened our scope to include both PPIE activities and studies employing qualitative methods. Qualitative methods are often used within applied health research to incorporate the voice of end users into the development and design of technologies, and therefore offer an insight into their views, preferences, and experiences.

### 2.2. Information sources

Systematic searches were being conducted across six key digital databases: Medline, Embase, PsycInfo, CINAHL, Scopus, and Web of Science. We searched for studies published up from inception of the databases up until May 2023.

#### 2.2.1. Search strategy

The search strategy was developed with the help of a [anonymized] librarian and incorporated key terms relating to digital technologies, PPIE, and neurological conditions:

### 2.3. Selection process

Using Rayyan, one reviewer (CW) conducted the initial title and abstract screening, the other four reviewers (MH, RM, AK & KB) then acted as second reviewers to 535 articles each for consistency. Full-text versions of these papers were retrieved and evaluated by four reviewers (CW, MH, RM & AK), with discrepancies resolved via a discussion or consultation with another reviewer. Reasons for exclusion were recorded as part of the screening process.

### 2.4. Data extraction and synthesis

To maintain rigor, data was extracted by one reviewer (MH) using a data extraction form. Data items for charting included: authors, year, aims/purpose, neurological condition, demographics (including sample size, age, sex, and ethnicity), geographical location, digital health technologies, PPIE activities, method of data collection and key findings. The timeframe precluded contacting authors for additional information. All qualitative data was then analyzed by one reviewer (MH), and results were reported in a narrative form.

#### 2.4.1. Interpretation of data

To create a focused analysis for both of our research objectives, we report the integration of PPIE activities and the impact of that PPIE as two distinct sections. The first section focuses on detailing the processes through which PPIE activities have been incorporated into the research cycle. We report on the collaborative efforts between researchers and contributors in shaping digital health technologies. The second section then focuses on examining the outcomes and effects from the integration of the PPIE activities.

### 2.5. Risk of bias assessment

As most studies returned qualitative explorations and the PPIE activities were descriptively reported, the National Institute of Health and Care Excellence (NICE) qualitative checklist was the tool we used to assess study quality [38]. This checklist facilitates rigorous evaluation of qualitative research and is commonly used to assess healthcare literature. Full qualitative appraisal is illustrated in Appendix A.

### 2.6. Development of recommendations

During the development of our reporting recommendations, we consulted an expert by experience (i.e. a former carer in dementia and Parkinson’s disease with PPIE experience). The recommendations cover recruitment of PPIE contributors for digital health research and include consideration of recruiting in a way that ensures inclusivity (i.e. digital exclusion). We must note that our contributor did not represent a population with such barriers. After sharing the results with the study team for feedback, we engaged in a discussion with a contributor and members of the review team including two with PPIE experience (MH, JL & RM). The discussion aimed to assess the current state of PPIE integration based on our results and to brainstorm recommendations for future researchers. Notes were taken during capturing core themes which then informed the development of recommendations. These recommendations underwent thorough review by all coauthors and the contributor, suggestions were made, and recommendations were altered to reflect the consensus view. Coauthors brought holistic expertise to the topic, i.e., PPIE, neurological conditions, qualitative analysis, and digital health.

## 3. Results

**Figure 1.**
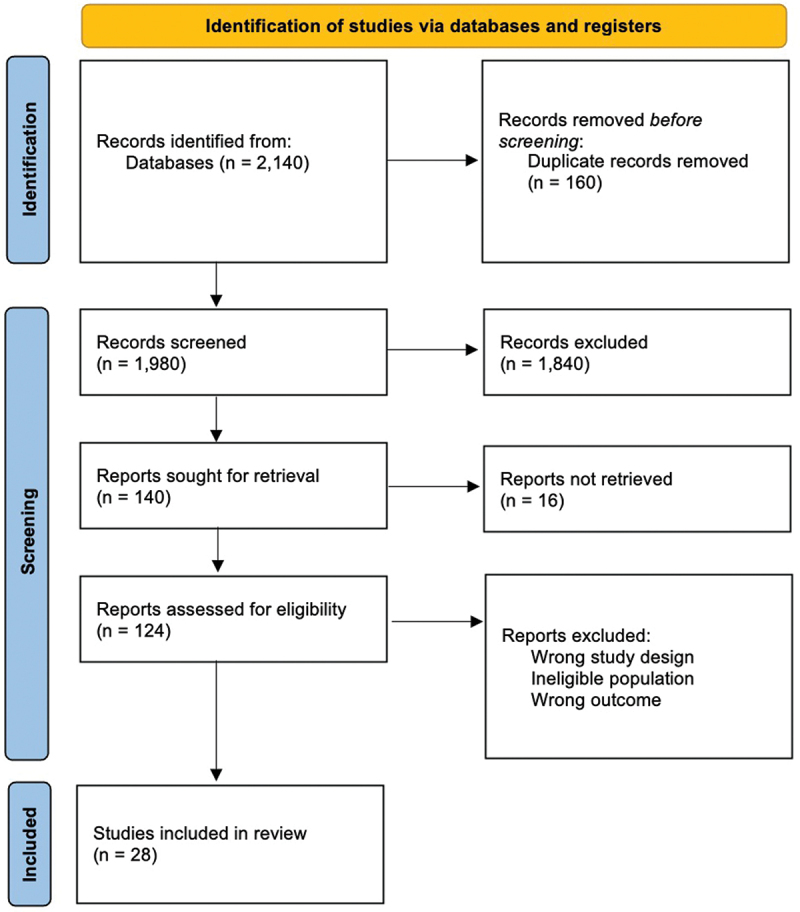
PRISMA flow diagram.

We searched six electronic databases for articles published before May 2023 and identified a total of 2,140 records. After removing duplicates, we screened 1,980 titles and abstracts. 124 articles were subjected to a full-text review based on the eligibility criteria, ultimately leaving us with 28 included publications. Full details of the article selection can be found in Figure 1. Regarding methodological quality, all included papers were appropriate (Appendix A).

Out of the 28 papers included, 25 were studies with a qualitative design (89%) and 3 were descriptive reports of PPIE activities (11%). The majority of papers involved people with neurological conditions (*n* = 24, 86%) and 15 involved carers of people with neurological conditions. A range of neurological conditions were reported on, but the majority were Alzheimer’s, cognitive impairment and related dementias (*n* = 11, 39%), and Parkinson’s disease (*n* = 6, 21%). Papers were mainly European (*n* = 22, 79%). Refer to Table 1 for the inclusion and exclusion criteria, and Table 2 for the detailed search strategy applied.

**Table 1.** Core inclusion/exclusion criteria.

Variable	Inclusion Criteria	Exclusion Criteria
Language	Published in the English language	Published in a language other than English
Publication time	Published up until May 2023	N/A
Topic	Qualitative methods or reports of PPIE activities that use the patient perspective in shaping digital health technologies	Publications that fail to include PPIE activities or use the patient perspective in shaping digital health technologies
Population	People who have been diagnosed with a neurological condition (Parkinson’s Disease, Huntington’s Disease, Multiple Sclerosis, Dementia, Alzheimer’s Disease, Epilepsy, Lou Gehrig’s Disease, Chronic Traumatic Encephalopathy, etc.)	People who have *not* been diagnosed with a neurological disease
Publication Type	Peer-reviewed publication Open Access	Study protocols Conference abstracts Reviews Meta-analyses Posters Presentation
Setting	Community-based research Home-based research Clinical-based research	N/A

PPIE = Patient and Public Involvement and Engagement.

**Table 2.** Search strategy.

	Combined with the ‘AND’ function				
Combined with the ‘OR’ function	Digital endpoints	Digital devices/fitness trackers/motion capture	Wearables	Monitoring/ambulatory	Digital assessment
	Patientparticipation	Patient voice	Patient engagement/public engagement	Public involvement/patient participation	Surveys and questionnaires/patient survey/focus groups/patient interviews
	Neurological disease/disorder	Neurology	Parkinson’s Disease, Huntington’s Disease, Multiple Sclerosis, Dementia, Alzheimer’s Disease, Epilepsy, Lou Gehrig’s Disease, Chronic Traumatic Encephalopathy		

In terms of demographics across papers, articles were published from 2015–2023. Age ranges varied, covering a variety of different age groups and both male and female participants were represented equally. Ethnicity was scarcely reported; however, there were participants represented from all continents in the few papers that reported this demographic information (see Table 3).

**Table 3.** Study characteristics.

Author & Year	Aims/Purpose	Condition	Participants			Geographical Location	DHT	PPIE	Method of Data Collection	Key Findings
			Sample Size	Age	Sex					
AlMadin et al. 2020	To identify the perspectives of healthcare professionals and patients linked to current assessment methods, and preferences and requirements of wearables	Parkinson’s disease	n = 12 PwPD	56-88 years	5 females 7 males	UK	Wearable devices	Exploring views/preferences via qualitative exploration	Focus groups	PwPD support wearable devices, they make assessments easier. No concerns were raised about visibility or data sharing, but PwPD want to co-design wearables.
Amini et al. [51] 2019	To develop and evaluate an integrated system capable of detecting falls and freezing of gait, provide visual cues oriented to a user’s position, and provide a range of communication	Parkinson’s disease	n = 15 PwPD	54-92 years	3 females 12 males	UK	Visual cue system incl. fall and freezing detection	Assessing functionality andperformance via qualitative exploration	Focus groups	Findings demonstrated viability of the remote system. All participants reported that visual cues were helpful to them.
Clague-Baker et al. [55] 2023	To identify the experiences and attitudes of people with myalgic encephalomyelitis toward heart-rate monitoring	Myalgicencephalomyelitis	n = 515 PwME	>18-90 years	448 females 67 males	International	Heart-rate monitoring devices (activity-tracking watches)	Exploring experiences/ attitudes via qualitative exploration	Survey	PwME emphasized a need for more bespoke devices. The devices were described as restrictive. Reported barriers included finance issues and lack of support.
Fisher et al. [56] 2016	To evaluate the acceptability of wrist-worn sensors following assessment, both brief and prolonged periods of wearing	Parkinson’s disease	n = 34 PwPD	50-86 years		UK	Wrist-worn sensors	Exploring experiences via qualitative exploration	Survey	Long-term monitoring with wrist-worn sensors is acceptable, including public settings. However, discomfort from strap design was reported along with concerns regarding data impact due to motion.
Gaugler et al. [57] 2021	To determine whether a remote activity monitoring system benefitted caregivers of people with Alzheimer’s disease or related dementias	Alzheimer’s disease and related dementias	n = 30 carers			US	Remote activitymonitoring system	Exploring usability experience via qualitative exploration	Semi-structured interviews	Responses reflected dementia carers changing perceptions of engagement with the remote activity monitoring system over time, as well as reasons why it was perceived beneficial or not.
Groussard et al. [39] 2018	To co-design a mobilize cognitive assistant to enhance the autonomy of people living with acquired brain injuries	Traumatic brain injury	n = 6 (3 PwTBI/3 carers)	30-50 years	3 males with TBI	Canada	Services assistance mobile and intelligent application	Participatory design study that looked to co-design the device	Focus groups	Contributors were involved during all development phases, leading to successful design, implementation, and evaluation. Overall, they were able to use the mobile service for a long period incl. more complex use, i.e. integrating the device in their habits.
Hassan et al. [58] 2017	To involve members of the public in discussions about acceptability & feasibility of multiple devices and research designs to inform the development of a device pool, software platform and written guidance to support future studies	Dementia and cognitive impairment	n = approx. 33	50-70+ years	11+ females 9+ males	UK	Multiple wearable devices	Involvement study that explores views/preferences of the public	Interactive workshops, drop-in sessions and meetings	Acceptability depended on ease of use, comfortability, and discreetness of the device. Recommendations included technical support and regular feedback to improve participation.
Infarinato et al. [60] 2020	To evaluate eWALL, a platform that monitors users’ physical and cognitive behavior, and provides feedback and motivation	COPD and mild cognitive impairment	n = 38 (23 PwCOPD/15 PwMCI)	*M* = 65.4 years (PwCOPD) *M* = 71.9 years (PwMCI)	15 females 23 males	Italy The Netherlands Denmark	A home care platform that monitors aspects of a user’s condition	Exploring acceptance and views via qualitative exploration	Semi-structured interviews	PwCOPD & MCI are open to this technology and are interested in insights about their condition via the interface.
Jacklin et al. [43] 2020	To explore the acceptability of the CareBand	Dementia	n = 29 incl. PwD, older adults, carers & care providers	45+ years		Manitoulin, Ontario	CareBand – a wearable location and activity monitoring device	Exploring views via qualitative exploration	Focus groups	Recommendations regarding aesthetics and functionality were suggested incl. general suggestions (e.g. size, style, incorporation of additional dementia-related symptoms, cost model) and suggestions specific to indigenous culture (e.g. environmental hardening of the hardware, language in messaging, support, land-based activities, wide support network.
Kearns et al. [59] 2022	To determine feasibility of the concept of a passive safety monitoring system for detecting cognitive impairment in electric powered wheelchair users	Cognitive impairment	n = 17 (9 PwCI/8 staff caregivers)		13 females 4 males	US	Passive safety monitoring system	Exploring views via qualitative exploration	Focus groups	This technology would be accepted by many; however concerns were raised regarding privacy, loss of autonomy, liability, and non-maleficent need for safety of users and peers.
Kenny et al. [49] 2022	To understand views and need of people with Parkinson’s regarding wearable devices for disease monitoring and management	Parkinson’s disease	n = 24 PwPD	53-84 years	10 females 14 males	Southern Ireland	Wearable devices	Exploring views/needs via qualitative exploration	Focus groups	Wearing the device was both feasible and acceptable if they were user-friendly. The devices must be used as a supplement to clinician-led health care. Concerns were shared about functionality rather than aesthetics.
Kettlewell et al. [46] 2018	To explore barriers and enablers for the uptake and use of ‘Brain in Hand’ (smartphone application) in clinical practice & identify potential adaptation of the app for use with people with Acquired brain injury	Acquired Brain Injury	n = 25 (20 ABI survivors/5 carers	18-51+ years	8 females 17 males	UK	‘Brain in Hand’ smartphone application	Exploring views via qualitative exploration	Piloting to adaptquestionnaire and focus groups	The questionnaire was adapted, based on contributor involvement, in a way that was more accessible and easier to complete by creating one for ABI survivors and carers, and another for professionals, they removed frequently unanswered or irrelevant questions. Comments made in the focus groups supplemented questionnaire material. Reported enablers included competency, personalization, and identifying perceived needs. Reported barriers included physical/cognitive inability to use a smartphone, potential cost and reliability of tech, and no desire to use tech or change from existing strategies.
Koopman et al. 2020 [44]	To create a blood pressure visualization EHR prototype that includes patient-generated blood pressure data	Hypertension	n = 16 PwH	18+ years		US	Blood pressure visualization EHR	Exploring views via qualitative exploration	Focus groups	Focus groups were used to iteratively test and refine the visualization in a series of 7 formative design focus groups, with 5 iterations to reach the final prototype. These groups identified needs and design elements that would satisfy needs (e.g. inclusion of annotations).
Lazarou et al. 2021 [48]	To explore human factors, needs, and requirements of people with dementia & caregivers with respect to supportive and interactive mHealth applications	Cognitive impairment	n = 31 (15 PwCI/16 carers)	*M* = 69.93 (PwCI) *M* = 52.53 (carers)	20 females 11 males	Greece	Mobile Health apps	Exploring views and needs via qualitative exploration	Patient involvement survey	All involved were likely to use mHealth, with desired features being the improvement of memory and cognition, assistance on medication treatment, and perceived ease of use. However, patients were concerned about privacy of their data.
Morgan-Jones et al. [47] 2020	To explore perceptions, motivators, and barriers relating to the adoption of wearable activity trackers for people with Huntington Disease, for monitoring and managing their lifestyle and sleep	Huntington Disease	n = 15 PwHD, carers and family		10 females 5 males	Bucharest Romania UK	Wearable activity trackers	Exploring views via qualitative exploration	Focus groups	Patients were more receptive to wearable trackers than the family members or carers. These trackers must be accurate, have a useful purpose, be easy/enjoyable to use, and be compatible with users’ lifestyles. It is important that the user understands the data generated from the tracker.
Stavropoulos et al. 2021	To capture preferences, priorities, and concerns of people with Alzheimer’s disease and their carers for using monitoring wearables	Alzheimer’s disease	n = 21 (11 PwCI/10 carers)			Europe	Monitoring wearables	A PPI activity with qualitative elements that looked at the development of digital assessment methods	Open discussionsand questionnaires	Participant feedback indicated that activity levels, heart rate/respiratory rate, and practical elements such as appearance, battery life, and the device being waterproof were all relevant aspects to consider in the development of wearable devices.
Costa Strutzel et al. 2019	To monitor patients with functional loss and to improve the support to caregivers’ communication with the health team professionals	Dementia	n = 38	*M* = 61	32 females 6 males	South America	Mobile system for elderly monitoring (SMAI)	Exploring views via qualitative exploration	Focus groups	The application provided participants with a sense of being a part of the health team. Overall, the SMAI functionalities were useful for caregivers.
Thomas et al. [42] 2021	To create a digital toolkit comprising of homework tasks (e.g. activity diary, goal planner, thought diary) of the FACETS program. To also consider end users’ unique perspectives throughout design, prototyping, and testing stages	Multiple Sclerosis	n = 11 PwMS	34-62 years	7 females 4 males	UK	Digital toolkit	Exploring views via qualitative exploration	Focus groups and semi-structured interviews	Participants felt the toolkit would be useful. Suggestions were made to improve design, functionality, and content, and some of these suggestions were implemented immediately (e.g. self-monitoring feedback such as graphs and dashboard).
Tierson et al. 2021	To investigate the functional, psychosocial, and environmental needs of people living with dementia and their caregivers toward the design and implementation of smart home systems	Dementia	n = 100 (46 PwD/54 carers)			UK	Smart home system	Exploring views via qualitative exploration	Semi-structured interviews, focus groups and workshops	The participatory design supported the triangulation of stakeholder perspectives to aid the development of a more patient-centered intervention and their translation to clinical practice and public health strategy.
Van Andel et al. [52] 2015	To describe stakeholders and their moral values that are relevant to the development and use of a seizure detectors, and to present four design choices incl. the corresponding risks and benefits in terms of the identified values	Epilepsy	n = 8 incl. PwE and carers			The Netherlands	Seizure detectors	Exploring views via qualitative exploration	Consultations and survey	The anticipated values, risks and benefits were used to inform the design of these devices. Carers mostly valued for the detector to be reliable, and operation of the detector ended up as the lowest priority.
VanWestrhenen et al. [61] 2021	To explore caregivers’ needs and wishes surrounding seizure detection devices	Epilepsy	n = 5 carers			The Netherlands	Seizure detectors	Exploring views via qualitative exploration	Focus groups	perspectives were explored so they could contribute to the design process of seizure detectors. Trust was identified as the most important factors, incl. providing insight on all parameters, personal adjustment to algorithms, recommendations by a neurologist, and a set-up period.
Virbel-Fleischman et al. [66] 2022	To evaluate the usability, user experience, and patients’ perceptions of body worn sensors	Parkinson’s disease	n = 22 PwPD	41-79 years	13 females 9 males	France	Body worn sensors	Exploring views via qualitative exploration	Semi-structured interviews	All expressed an interest in body worn sensor monitoring; PwPD envisioned that this device could help improve their symptoms. They anticipated that this device would assist monitoring in clinical practice, be useful for treatment adjustment and supportive for research.
Voigt et al. [40] 2020	To develop a pathway-based care model and corresponding patient portal for multiple sclerosis patients and HCPs, as a digital tool to deliver the care model	Multiple sclerosis	n = 222 incl. PqMS, confidants and informal caregivers	18-80+ years		Germany	Patient portal	Exploring views via qualitative exploration	Survey and workshops	The portal was determined to most likely be a useful, high-quality electronic health tool for patient management. Patient voices were used in the development of this tool.
Wendrich &Krabbenbord [63] 2023	To explore how digital self-monitoring becomes materialized in the everyday lives of patients with multiple sclerosis	Multiple sclerosis	n = 26 PwMS	28-64 years	17 females 9 males	The Netherlands	Digital self-monitoring technology	Exploring views via qualitative exploration	Semi-structured interviews	PwMS did not perceive digital self-monitoring as useful for their self-management and thought it as an inconvenience to perform self-monitoring tasks. They also anticipated an emotional burden of being reminded that they had MS due to the continuous self-monitoring.
Wrede et al. [62] 2021	To explore the expected benefits, barriers, needs, and requirements toward unobtrusive in-home monitoring from the perspective of formal and informal carers of community-dwelling people with Dementia	Dementia	n = 35 carers (19 informal/16 formal)	*M* = 60.5 (informal carers) *M* = 39.2 (formal cares)	16 females 3 males	The Netherlands	In-home monitoring	Exploring views via qualitative exploration	Semi-structured interviews and focus groups	All caregivers regarded in-home monitoring as a support tool that could contribute to a shift from reactive to more preventative and proactive care. The systems were expected to inform about falls, day and night rhythm, personal hygiene, nocturnal restlessness, and eating/drinking habits.
Wrede et al. [45] 2022	To explore the value proposition of UM technologies in home-based dementia care, and preconditions for successful implementation from a multi-stakeholder perspective	Dementia	n = 9 (4 PwD/5 carers)	76-82 years (PwD) 42-77 (carers)		The Netherlands	In-home monitoring	Exploring views via qualitative exploration	Semi-structured interviews and focus groups	This tech was identified as a tool that could help facilitate personalized timely care, which may help save caregivers’ resources.
Ozanne et al. [64] 2018	To explore perceptions regarding use of wearable technology in disease monitoring/management in Parkinson’s and epilepsy	Parkinson’s disease and epilepsy	n = 25 (15 PwPD/10 PwE)	48-80 (PwPD) 22-56 (PwE)	10 females 15 males	Sweden	Wearable devices	Exploring views via qualitative exploration	Focus groups	To use wearables, patients must be well-informed and find an added value in using them. They must be user-friendly, attractive and show clinical efficacy in improving disease management.
Peeters et al. [65] 2021	To identify the values, needs, and attitudes of multiple stakeholders regarding wearables in the care of people with dementia and challenging behavior	Dementia	n = 24 (7 PwD/17 carers)			The Netherlands	Wearable devices	Exploring views via qualitative exploration	In-depth interviews & focus groups	When used by carers, the wearables were accepted as a supportive tech in the care of people with challenging behavior. On an individual level, the tech improvement needs to be made to the design of the device and information about technology to enhance acceptability to patients.

PPIE = Patient and Public Involvement and Engagement.

DHT = Digital Health Technologies.

PwPD = People with Parkinson’s disease.

PwME = People with myalgic encephalomyelitis.

PwBI = People with traumatic brain injury.

PwCOPD = People with chronic obstructive pulmonary disease.

PwMCI = People with mild cognitive impairment.

PwD = People with dementia.

PwCI = People with cognitive impairment.

ABI = Acquired Brain Injury.

PwMS = People with Multiple Sclerosis.

PwH = People with Hypertension.

PwE = People with Epilepsy.

PwHD = People with Huntington’s disease.

### 3.1. Synthesis of results

This section is divided into two parts: (1) the integration of PPIE on the development of digital health technologies and (2) the impact of these strategies.

#### 3.1.1. The integration of PPIE

All studies involved people with neurological conditions and/or their carers in shaping the development of digital health technologies, whether that be through researcher-led qualitative studies or collaborations with contributors in patient/public-led discussions and activities. We observed various forms of involvement during three key research phases (design, development, and implementation), such as collaborative efforts in study design (39%), exploration of end users’ views and acceptability (71%), and active engagement in usability testing (21%).

##### 3.1.1.1. Design

Eleven papers incorporated public and patient contribution into the design of digital health technologies [39–49]. Contributions mainly fell within 1) identifying relevant issues by exploring patient and carer needs and 2) assisting in the development of research questions or protocols [39–45,49]. Three papers utilized patient perspectives to guide the selection of, and adjustments to, the materials used, which were reported as enhancing the meaningfulness of their study design [46–48]. One paper included PPIE-related work (i.e. an acceptability questionnaire) that was developed in previously published work that was not directly linked to the activities in the reported study [47]. Although PPIE in this instance is not directly linked to the study reported, they do consider the value of materials developed with contributors.

##### 3.1.1.2. Development

Twenty papers demonstrated contributors actively contributing to the refinement of digital health technologies [40,42,43,50–66]. This included informing product design via designing graphical interfaces, providing feedback on prototypes, suggesting refinements to the tools, and producing user requirement recommendations [40,42,50–53]. The most apparent way in which contributors were involved in the development phase was through acceptability and feasibility explorations. Contributors reported their views and opinions on whether they would use the technologies under investigation, this included the usefulness and fit of these into the contact of their everyday lives [43,53–66]. This approach aimed to ensure digital health technologies were intuitive, user-friendly, culturally sensitive, and aligned with the users’ needs.

##### 3.1.1.3. Implementation

During the implementation phase (i.e. when the tools are tested by contributors), contributors played a role primarily in the evaluation of digital health technologies. Six papers included contributors in usability and clinical evaluations [41,42,51,54,57,63]. They provided feedback on functionality, perception of experience, appropriateness/acceptability of design and content, and ease of use. In some cases, this contributed to iterative improvements in their digital tools, e.g., one study re-designed their cognitive testing app, altering puzzles to gradually increase in difficulty and to allow participants to pause tasks and repeat instructions because of contributor feedback [41].

In comparing papers, we identified both similarities and differences in the integration of PPIE, which varied across publications. Most encompassed a researcher-led approach [40–48,50–52,54–57,59–66] where there was a single discrete input from contributors and work that was done *for* them rather than *with* them. Contrastingly, four of the papers embraced more of a collaborative, in-depth approach, which represented more of a partnership as the contributors formed part of the research team and guided elements of the study [39,49,53,58]. Thus, whilst the integration of PPIE remains a consistent theme, based on our current definition of PPIE, clear variability exists in the type, extent, depth, and quality of involvement. Another interesting similarity that we observed across papers was regardless of whether authors were reporting PPIE work or conducting qualitative research, they all referred to contributors as ‘participants’ or ‘subjects.’ This was expected in the qualitative studies; however, this was an unexpected finding to arise from the descriptions of PPIE work.

#### 3.1.2. The impact of PPIE

Overall, working with contributors was felt to provide valuable input in the development and refining of digital health technologies. Some contributors expressed a desire to assist in the co-design of these tools because it was felt by the contributors that this involvement would increase their receptiveness toward the devices [50]. Contributor input led to modifications (e.g. simplifying screen flow, using location-based services, displaying personalized information, altering tasks) that would ensure more patient-centered and inclusive approaches [41,42,44].

Further, contributor input in the qualitative studies demonstrated the potential to increase face validity of digital health technologies and to enhance their applicability in real-world contexts. A common theme that emerged was how these tools could be improved to increase patient acceptability. This included advice, such as including provision of visual cues, altering current designs to be more comfortable and less restrictive, focusing on improving aesthetics, making the devices more discreet, and offering education on the use of the technology [47,51,55,56,58,65]. One of the included studies demonstrated how PPIE could assist with educating users on digital health technologies, showing success in helping users grasp both simple and complex use of a mobile cognitive assistant [39]. Contributors also shared important functions that would be useful to them, enlightening researchers on how to design these tools with features that matter to the end user. This included suggestions such as measures to improve memory and cognition for patients with a cognitive impairment [48], including self-monitoring feedback with graph visualizations for patients with multiple sclerosis [42], and producing information for carers about patient’s falls, day and night rhythm, personal hygiene, nocturnal restlessness, and eating/drinking habits for patients with dementia [62].

Lastly, utilizing the patient and public voices had positive outcomes for literature contributions that could be used to underpin future research in the development of digital health technologies. Many of the included studies provided recommendations on how these tools could be improved from the perspective of the end user. Such recommendations included readily available tech support and regular feedback [58], and how aesthetic and functionality can be altered to be more specific to indigenous culture and rural life [43]. Overall, such involvement yielded insightful perspectives into making digital health technologies more acceptable for the target population, demonstrating both immediate impact via descriptions of existing iterative designs and future impact via reporting contributors’ insights on how to improve these tools with the end user in mind.

## 4. Discussion

PPIE is a recognized important component of the research process [26,67], particularly in neurological research. As digital health research gains increasing momentum in the neurological space, it is essential to understand how PPIE is being conducted and identify key learnings to optimally involve contributors across the research cycle in this area. This rapid review is the first to our knowledge to evaluate the integration and effectiveness of PPIE activities in the development of digital health technologies for neurological conditions. Key findings indicate that involvement is conducted sporadically across the research cycle, with little consistency in PPIE approaches, although we recognize that publishing constraints (i.e. word count) could mean that PPIE activities are underreported. In this review, contributors were mainly involved in the development research stage, and seldom involved in implementation. However, good examples of involving contributors in evaluating the appropriateness and acceptability of design are highlighted. Based on our results and through discussion and reflection with an experienced PPIE contributor and research experts in Parkinson’s disease, PPIE, qualitative research, and digital health, we have developed recommendations for the incorporation of PPIE in digital health research for neurological conditions.

A few recently published reviews [32–34] revealed the infrequent involvement of PPIE in healthcare research. This review included 28 papers involving qualitative research that looked at improving the service, development, or implementation of digital health technologies and reports of PPIE activities, as defined by the NIHR [27], with a focus on neurological research. This decision was made due to the scarcity of published evidence documenting PPIE-specific work. The most common publications were qualitative research studies, with only three being descriptions of PPIE activities. Therefore, we can confirm that PPIE is starting to be acknowledged as an important addition to this research field; however, there is still a lack of reporting when contributors are included as part of the research team rather than simply being seen as participants whose opinions are sought. This is further emphasized by only a few papers involving contributors at multiple stages, most including one-off contributions. Though we revealed a growing trend toward the integration of PPIE, we must continue to encourage continuous involvement of contributors in the research process. If we continue as we are, we risk developing ineffective digital health technologies; working in partnership with patients, carers, and members of the public can educate researchers, help them to identify relevant concerns, create appropriate research designs, and ultimately develop applicable tools that are much more likely to be useful to the end user [68].

The included studies demonstrated how the patient voice was instrumental in driving modifications to digital health technologies, fostering a more patient-centered and inclusive approach to measuring health remotely. Additionally, listening to views on acceptability and feasibility showcased the potential to enhance face validity and real-world applicability of digital health technologies, both with immediate effects on design iterations and future recommendations. These findings confirm benefits previously anticipated by including the patient voice, emphasizing the value of PPIE [27]. As most papers were research studies, impact was established through data analysis and publication outputs, which is commonly how impact is assessed [69]. This made it challenging to discern the precise influence of PPIE on each study, therefore we settled on the impact to research outcomes or what the authors had concluded. It is crucial that we begin to separate qualitative research from PPIE to accurately assess the impact of contributor involvement on the research process. Through clear differentiation, researchers can effectively evaluate the impact of PPIE activities in shaping research and thus understand the level of influence PPIE has.

We must also disentangle qualitative research and PPIE into two separate, clearly distinguishable entities, particularly as some research and PPIE activities are easily blurred, e.g., acceptability explorations. This would ensure that the unique involvement of contributors is appropriately recognized, as being a participant vs a PPIE advisor is different on many levels. A contributor’s role is much more active, working *with* the research team, giving advice, and being engaged in the decision-making processes [70]. They have a significant influence on the overall direction of the research, unlike participants whose involvement is usually limited to specific activities outlined in the protocol such as taking part in an interview. Acknowledging these differences recognizes the significant input put in by the contributors.

### 4.1. Recommendations for reporting of PPIE

Our findings demonstrate the need for more guidance on the reporting of PPIE, so researchers can become more informed on what to consider when incorporating PPIE into their digital health research. As an expert by experience, we worked alongside one PPIE contributor to develop recommendations. Key focal points included recognizing contributors as integral within the research team because of their expertise in lived experience, emphasizing their influence on research direction. Moreover, we discussed the importance of documenting this impact and acknowledging their contributions throughout. We spoke of the need for iterative, frequent, and early involvement of contributors, ensuring their expectations are met through transparent communications and discussing any proposed changes. Then, we discussed that minimum reporting recommendations would be useful for authors looking to integrate PPIE into their work to combat concerns regarding the word count restrictions in published articles. Although recommendations were considered specifically for digital health research, discussions highlighted the universality of PPIE principles. Fundamentally, PPIE should add meaningfulness to research in a manner appropriate to your contributors [27]. We propose the following to enhance the integration of PPIE with the aim to guide researchers in effectively involving contributors and ensuring inclusivity, transparency, and relevance throughout the research process:

#### 4.1.1. Refrain from labelling contributors as ‘participants’

researchers should acknowledge PPIE contributors as collaborators and specialists, admitting them as experts in their experience and refrain from labeling them as ‘participants.’ In this review, we see a prevalent label of ‘participants,’ even within the PPIE descriptions. This oversight undermines their valued expertise and insights, as contributors should be acknowledged and recognized [71]

#### 4.1.2. Ensure early and ongoing involvement

Researchers should ensure that PPIE contributors are involved at most, if not all, stages of the research process. We must engage contributors beyond a single timepoint. Further, PPIE should be considered early in the project lifecycle, preferably during the grant writing stage to ensure the project is relevant and meaningful from the outset. If this is not possible, PPIE should be incorporated at the study planning stages, allowing them to help shape the research agenda and methodology. Early engagement allows for effective utilization of contributors’ time [72].

#### 4.1.3. Clearly report PPIE impact

The impact of the PPIE activities should be reported clearly within papers. Transparent reporting of how PPIE has influenced the research is required, e.g., shaping research questions and designs, to informing methodology, and influencing design iterations. Acknowledging these changes through an impact log to evaluate the PPIE outcomes [73] and highlighting the process evolution will make it easier for researchers to determine the impact of PPIE and help them evaluate these strategies [74].

#### 4.1.4. Developing resources for researchers

Considerations must be had into developing resources, such as toolkits and case studies, to support researchers in implementing effective PPIE practice. Guidelines exist; however, these may be longer and inappropriate for short summaries/word counts (e.g. GRIPP2 reporting checklist) [74]. If researchers are struggling with limited reporting space, minimum reporting recommendations should be adhered to. This involved outlining key aspects such as recruitment, involvement stages, and impact on research. Table 4 sets out the minimum reporting guidelines:

**Table 4.** Minimum reporting guidelines.

Points to report	Example
Who was involved in the PPIE?	‘We consulted two PPIE contributors, including one healthcare professional and one expert by experience.’
What was their experience with the subject matter?	‘One contributor, who was well-versed in digital health tools, contributed experience in implementing health technologies within a healthcare setting. Our expert by experience, with prior involvement in PPIE activities, provided valuable insights into user perspectives of the implementation of digital health technologies.’
How did you conduct your PPIE? What did you ask of your contributors?	‘In conducting our PPIE, we used a participatory approach, involving our contributors in multiple stages of the research process, including development of the research question, designing the digital tool, and testing the appropriateness of the design. We did this through interactive workshops where the contributors could contribute feedback which led to iterations of the digital tool’
What experiences did your contributors share with you?	‘Our contributors highlighted the importance of designing digital tools with the target population in mind and offered solutions specifically tailored to the populations needs, e.g. allowing participants with dementia to repeat task instructions.’
How did this impact or change the research?	‘These insights drove the evolution our digital tool. Initially, we incorporated tasks that did not allow participants to reference instructions once pressing ‘go.’ However, in response to our contributors’ feedback, we modified this to enable participants to repeat task instructions, ensuring a more user-friendly experience’

#### 4.1.5. PPIE in digital health research

for this research area specifically, we recommend that researchers address common barriers including digital literacy and access considerations. Addressing this in recruitment of PPIE collaborators will ensure differing insights.

#### 4.1.6. Recognise the universality of PPIE principles

PPIE reporting is consistent across disciplines, though some additional principles may need to be considered, such as the previous recommendation suggests. Fundamentally PPIE should add meaningfulness to research in a manner appropriate to your contributors.

#### 4.1.7. Requirement for standardised terminology

Currently, there is no global standard definition of PPIE. We have adopted a well-established but UK-focused definition. The NIHR’s definition of PPIE is mature, having been an integral part of its establishment in 2006. Different countries use varying terminology. To accurately determine the global state of PPIE inclusion, we must first identify universally applicable PPIE principles and create a clear, globally accepted definition with standardized terminology. Once this is achieved, we can conduct a re-review to deepen our understanding of international perspectives, which will enhance our understanding and help improve PPIE practices worldwide.

### 4.2. Strengths and limitations

This rapid review is the first that has examined existing PPIE work conducted in digital technology research with a focus on neurological conditions. Strengths include the use of a comprehensive and systematic search strategy to minimize the risk of missing and preregistered inclusions. Additionally, recommendations for reporting PPIE in digital health research were co-developed by a multi-disciplinary team including a PPIE contributor, with significant experience contributing to advisory panels, grant reviews and monitoring research studies, alongside researchers with expertise in PPIE, qualitative research, digital health, and various neurological conditions. While efforts were made to capture all expertise among the research team, in future a broader team may be able to add additional perspectives. For example, there would be added value in incorporating more experts by experience, such as contributors with different neurological conditions.

A significant limitation of this study was our incorporation of qualitative research in our results synthesis, as this is not consistent with PPIE as defined by NIHR but was recommended due to the lack of PPIE activities reported. This highlights the current parity of PPIE reporting in the field, which our review recommendations hope to rectify. Additionally, as this is a rapid review, potentially relevant studies may have been missed as we did not contact authors for additional information, screen reference lists of the included studies or seek gray literature, non-English language studies, or conferences abstracts. Future research could address this limitation by conducting a systematic review instead.

A final limitation regards the search strategy used to identify articles. As we have taken on a UK-focused definition of PPIE based on NIHR and with this being a rapid review, search terms only incorporated this terminology. However, other countries use slightly different terms which may not have come out in the search. For example, Australia uses ‘consumer research’ and U.S.A. uses ‘partnering with research participants’ or ‘community participation’ generally. However, these approaches do differ slightly from the approaches typically used in the UK, e.g., in Australia, people don’t get renumeration, whereas the NIHR value this [75]. Future research could expand the scope of terminology to ensure a more comprehensive analysis of the integration of PPIE across different countries.

## 5. Conclusion

This review revealed limited integration of PPIE in the development of digital health technologies for neurological conditions. Recognizing the scarcity of published evidence, we included both qualitative research and PPIE-specific work. Despite the importance of qualitative research in shaping digital technology development, we must emphasize the need to distinguish between these two entities to ensure clarity and appreciation. Moving forward, it is crucial that researchers begin to involve PPIE contributors and report their involvement and impact transparently. Therefore, we propose a set of recommendations and by embracing them, researchers can effectively involve contributors, ensuring inclusivity, transparency, and relevance in the development of digital health technologies. A future review can then be undertaken when additional studies are published in this area, to see if the recommendations made in this paper have been implemented.

## Supplementary Material

Appendix A.docx

Appendix B PRISMA_2020_checklist.docx

## Data Availability

Data sharing is not applicable to this article as no new data were created or analyzed for this study.

## Article highlights

- Limited integration of PPIE: This study revealed inconsistent integration of PPIE in the development and implementation of digital health technologies for neurological conditions, with most involvement limited to development stages.
- Varied contributor roles: Across articles, contributors played various roles, from influencing study design and providing feedback on digital tools to participating in usability testing. The depth of this involvement varied signficantly.
- Impact on digital health tools: Contributor feedback led to modifications in digital health technologies, ensuring more patient-centered designs by improving usability, personalisation, and acceptability of the tools in real-world settings.
- Need for better reporting: This study demonstrates the need for standardised and transparent reporting of PPIE and its impact in digital health research, recommending continous involvement and recognition of contributors as active research partners rather than participants.
