# Supplementary material for: How can patients shape digital medicine? A rapid review of patient and public involvement and engagement in the development of digital health technologies for neurological conditions
Source: Expert Rev Pharmacoecon Outcomes Res. 2024 Oct 7;25(2):137–54. doi: 10.1080/14737167.2024.2410245 (PMC11789707; doi:10.1080/14737167.2024.2410245)
Supplement: Appendix A.docx [file IERP_A_2410245_SM1497.docx]

Appendix A: National Institute of Health and Care Excellence (NICE) qualitative checklist

| **Author** | **Theoretical Approach** | | **Study Design** | **Data Collection** | **Trustworthiness** | | | **Data Analysis** | | | | | | **Ethics** | **Overall Assessment** |
| --- | --- | --- | --- | --- | --- | --- | --- | --- | --- | --- | --- | --- | --- | --- | --- |
|  | Is a qualitative approach appropriate? | Is the study clear in what it seeks? | How defensible rigorous is the research design methodology? | How well was the data collected? | Is the role of the researcher clearly defined? | Is the context clearly described? | Were the methods reliable? | Is the data analysis sufficiently rigorous? | Is the data 'rich'? | Is the analysis reliable? | Are the findings convincing? | Are the findings relevant to the aim of the study? | Is there adequate discussion of any limitations encountered? | How clear and coherent is the reporting of ethics? | As far as can be ascertained, how well was the study conducted? |
| AlMahadin, G., et al. (2020) | + | + | + | + | + | + | + | + | + | + | + | + | + | + | ++ |
| Amini, A., et al. (2019) | + | - | - | - | - | - | - | - | - | ? | - | - | - | + | - |
| Clague-Baker, N., et al. (2023) | + | + | + | + | - | + | - | + | + | - | + | + | + | + | + |
| Fisher, J. M., et al. (2016) | + | + | + | + | + | + | + | + | + | + | + | + | + | + | ++ |
| Gaugler, J. E., et al. (2021) | + | + | + | + | + | + | + | + | + | + | + | + | + | + | ++ |
| Groussard, P. Y., et al. (2018) | + | + | + | + | + | + | + | + | + | + | + | + | + | + | ++ |
| Hassan, L., et al. (2017) | + | + | + | + | + | + | + | + | + | - | + | + |  |  |  |
| Infarinato, F., et al. (2020) | + | + | + | + | + | - | + | + | - | + | - | + | + | + | + |
| Jacklin, K., et al. (2020) | + | + | + | + | + | + | - | + | + | + | - | + | + | + | ++ |
| Kearns, W. D., et al. (2022) | + | + | + | + | + | + | + | + | + | + | - | + | + | + | ++ |
| Kenny, L., et al. (2022) | + | + | + | + | + | + | + | + | + | + | + | + | + | + | ++ |
| Kettlewell, J., et al. (2018) | + | + | + | + | - | + | + | - | + | - | + | + | + | + | + |
| Koopman, R. J., et al. (2020) | + | + | + | + | + | + | + | + | + | + | + | + | + | + | ++ |
| Lazarou, I., et al. (2021) | + | + | + | + | - | + | + | - | + | - | - | + | - | + | + |
| Morgan-Jones, P., et al. (2022) | + | + | + | + | + | + | + | + | + | + | + | + | + | + | ++ |
| Stavropoulos, T. G., et al. (2021) | + | + | + | + | + | + | + | - | + | - | - | + | - | + | + |
| Costa Stutzel, M. C., et al. (2019) | + | + | + | + | + | + | + | - | + | - | + | + | - | + | + |
| Thomas, S., et al. (2021) | + | + | + | + | + | + | + | + | + | + | + | + | + | + | ++ |
| Tiersen, F., et al. (2021) | + | + | + | + | + | + | + | + | + | + | - | + | + | + | ++ |
| Van Andel, J., et al. (2015) | + | + | + | + | + | + | - | - | + | + | - | + | + | + | + |
| van Westrhenen, A., et al. (2021) | + | + | + | + | + | + | + | + | + | + | + | + | + | + | ++ |
| Virbel-Fleischman, C., et al. (2022) | + | + | + | + | + | + | + | + | + | + | + | + | + | + | ++ |
| Voigt, I., et al. (2020) | + | + | + | + | - | + | + | - | + | - | + | + | + | - | - |
| Wendrich, K. and Krabbenborg, L. (2023) | + | + | + | + | + | + | - | + | + | + | + | + | + | + | ++ |
| Wrede, C., et al. (2021) | + | + | + | + | - | + | + | + | + | + | + | + | + | + | ++ |
| Wrede, C., et al. (2022) | + | + | + | + | - | + | + | + | + | + | + | + | + | + | ++ |
| Ozanne, A., et al. (2018) | + | + | + | + | + | + | + | + | + | + | + | + | + | + | ++ |
| Peeters, M. W. H., et al. (2021) | + | + | + | + | + | + | + | + | + | + | + | + | + | + | ++ |
